# Poloxamer 188 Attenuates Ischemia-Reperfusion-Induced Lung Injury by Maintaining Cell Membrane Integrity and Inhibiting Multiple Signaling Pathways

**DOI:** 10.3389/fphar.2021.650573

**Published:** 2021-07-15

**Authors:** Shih-En Tang, Wen-I Liao, Hsin-Ping Pao, Chin-Wang Hsu, Shu-Yu Wu, Kun-Lun Huang, Shi-Jye Chu

**Affiliations:** ^1^ Institute of Aerospace and Undersea Medicine, National Defense Medical Center, Taipei, Taiwan; ^2^ Department of Internal Medicine, Tri-Service General Hospital, National Defense Medical Center, Taipei, Taiwan; ^3^ Department of Emergency Medicine, Tri-Service General Hospital, National Defense Medical Center, Taipei, Taiwan; ^4^ The Graduate Institute of Medical Sciences, National Defense Medical Center, Taipei, Taiwan; ^5^ Department of Emergency and Critical Medicine, Wan Fang Hospital, Taipei Medical University, Taipei, Taiwan

**Keywords:** acute lung injury, ischemia-reperfusion, poloxamer 188, hypoxia/reoxygenation, membrane integrity

## Abstract

**Background:** Poloxamer 188 (P188) possesses anti-inflammatory properties and can help to maintain plasma membrane function. P188 has been reported to exert beneficial effects in the treatment of various disorders. However, the effects of P188 in ischemia/reperfusion (IR)-induced acute lung injury have not been examined.

**Methods:** We investigated the ability of P188 to attenuate IR-induced acute lung injury in rats and hypoxia/reoxygenation (HR) injury in murine epithelial cells. Isolated perfused rat lungs were exposed to 40 min ischemia followed by 60 min reperfusion to induce IR injury.

**Results:** IR led to lung edema, increased pulmonary arterial pressure, promoted lung tissue inflammation and oxidative stress, and upregulated the levels of TNF-α, IL-6 and CINC-1, and increased Lactic dehydrogenase (LDH) activity in bronchoalveolar lavage fluid. IR also downregulated the levels of inhibitor of κB (IκB-α), upregulated nuclear factor (NF)-κB (NF-κB), and promoted apoptosis in lung tissues. P188 significantly suppressed all these effects. *In vitro,* P188 also exerted a similar effect in murine lung epithelial cells exposed to HR. Furthermore, P188 reduced the number of propidium iodide-positive cells, maintained cell membrane integrity, and enhanced cell membrane repair following HR.

**Conclusion:** We conclude that P188 protects against lung IR injury by suppressing multiple signaling pathways and maintaining cell membrane integrity.

## Introduction

The nonionic triblock co-polymer poloxamer 188 (P188; molecular weight 8.4 kDa; also known as Pluronic® F68) consists of two hydrophilic side-chains attached to a central polyoxypropylene molecule. P188 has been shown to reduce cell membrane damage and cell injury in various *in vivo* and *in vitro* models (Moloughney and Weisleder, 2012; Zarrintaj et al., 2020), and employed as an antithrombotic drug, a rheological agent in sickle cell disease, and an emulsifying agent in artificial blood (Moloughney and Weisleder, 2012; Zarrintaj et al., 2020). Most importantly, P188 has been demonstrated to exert plasma membrane-sealing properties, which enhance the repair of skeletal muscle cells, cardiac myocytes, neurons, fibroblasts, and corneal endothelial cells after a variety of insults (Moloughney and Weisleder, 2012). P188 also protected neurons from injury induced by spinal cord compression, excitotoxicity, traumatic brain injury, and acute intracranial hemorrhage (Bao et al., 2012; Wang et al., 2015; Paleo et al., 2020). Furthermore, several studies have indicated that P188 can protect against intestinal, muscle, myocardial, cerebral, and hepatic ischemia-reperfusion (IR) injury, and prolong the survival of cardiac, renal, and skin allografts (Forman et al., 1992; Hunter et al., 2010; Yildirim et al., 2015; Bartos et al., 2016). P188 has been suggested to exert these protective effects by intercalating with lipid bilayers to restore the integrity of damaged plasma membranes, and also by protecting cells against apoptosis and oxidative stress (Moloughney and Weisleder, 2012; Gu et al., 2013).

Lung transplantation is the only effective therapy for patients with advanced lung disease (Laubach and Sharma, 2016). Exposure of the donor lung to ischemia for up to several hours is unavoidable during the transplantation procedure, and the severity of lung malfunctions correlates with the duration of ischemia. Early reestablishment of the blood supply to the lung limits the severity of ischemia. However, reperfusion of the transplanted lung can induce an intense inflammatory response and cellular alterations that further exaggerate tissue injury and cause cell death, and these changes may subsequently result in permeability lung edema (Kalogeris et al., 2012; Laubach and Sharma, 2016). Even with the enormous improvements in lung transplantation procedures, IR-induced lung injury remains the major cause of primary graft dysfunction and early recipient death after lung transplantation. IR-induced lung injury also contributes to the progression to chronic lung allograft dysfunction (Laubach and Sharma, 2016).

The pathogenesis of IR-induced lung injury involves a spectrum of pathological processes that result in the activation of inflammatory response genes. For example, alveolar epithelial cells exposed to IR release inflammatory cytokines, and this response is subsequently magnified that result in plasma membrane disruption (Laubach and Sharma, 2016). Damaged alveolar resident cells are observed in experimental models of lung IR and human lungs with acute respiratory distress syndrome (Kalogeris et al., 2012; Laubach and Sharma, 2016). Thus, it is reasonable to speculate that cell injury and repair contribute to the pathogenesis of IR-induced lung injury (Cong et al., 2017).

Loss of plasma membrane integrity induces various downstream inflammatory events and exacerbates cell injury during IR and represents a major pathogenic mechanism that leads to lung edema and epithelial cell death (Kalogeris et al., 2012). A previous study showed that P188 reduced ventilator-induced lung injury in isolated perfused rat lungs by restoring plasma membrane integrity and protecting alveolar resident cells from stress-induced necrosis (Plataki et al., 2011). In addition, P188 has been reported to possess anti-inflammatory properties (Moloughney and Weisleder, 2012). Strategies that maintain or restore the integrity of cell membranes exposed to IR and suppress the associated inflammatory response may represent a potential treatment approach for IR-induced injury. In this study, we examined the effect of P188 on IR-induced lung injury and explored the mechanisms by which P188 maintains cell membrane integrity and inhibits the inflammatory response.

## Materials and Methods

### Isolated Perfused Rat Lung Model

Sprague-Dawley male rats weighing 350 ± 20 g were handled according to the guidelines of the National Institutes of Health; all animal experiments were approved by the Animal Review Committee of the National Defense Medical Center (Permit Number: IACUC-18-218). Rat lungs were isolated and perfused in the chest as previously described (Chu et al., 2002; Wu et al., 2015; Wu et al., 2017). Briefly, after tracheotomy, the rats were ventilated with air containing 5% CO_2_ at 60 breaths/min at a tidal volume of 3 ml with a positive end-expiratory pressure of 1 cm H_2_O. A sternotomy was performed, heparin was injected into the right ventricle, and approximately 10 ml intracardiac blood was collected. The pulmonary artery was cannulated, and a drainage cannula was placed in the left ventricle. The cannulae were connected to the perfusion circuit and perfused with physiological salt solution (119 mM NaCl, 4.7 mM KCl, 1.17 mM MgSO_4_, 22.6 mM NaHCO_3_, 1.18 mM KH_2_PO_4_, 1.6 mM CaCl_2_, 5.5 mM glucose, 50 mM sucrose) containing 4% bovine serum albumin. The 10 ml collected blood was added to the perfusate and subsequently mixed with the physiological salt solution as a perfusate for the isolated lungs. The roller pump (Minipuls 2; Gilson Medical Electronic, Middleton, WI, United States) was maintained at a flow rate of 8–10 ml/min. *In situ* isolated rat lungs were placed on an electronic scale to monitor real-time changes in lung weight. Left atrial pressure, which indicates pulmonary venous pressure (PVP), and the pulmonary artery pressure (PAP) were constantly recorded through the side arm of the cannula using pressure transducers (Gould Instruments, Cleveland, OH, United States).

### Microvascular Permeability Assay

K_f_, an indicator of microvascular permeability to water was estimated from the change in lung weight due to elevated venous pressure, as described previously (Wu et al., 2015; Wu et al., 2017). K_f_ was designated as the initial weight gain rate (g min^−1^) divided by the PVP (10 cmH_2_O) and lung weight, and expressed in units of g min^−1^ cm H_2_O^−1^ × 100 g.

### Determination of Lung Weight/Body Weight and Wet/Dry Weight Ratios

After IR, the right lung was removed from the hilar region and LW was computed to determine the LW/BW ratio. A part of the right upper lobe of the lung was harvested from each rat, weighed, and placed in an oven at 60°C for 48 h. The dry weight was determined, and the W/D lung weight ratio was calculated.

### Total Cell Counts, LDH Activity, and Levels of Protein, Cytokine-Induced Neutrophil Chemoattractant-1, Interleukin-6, and Tumor Necrosis Factor-α in Bronchoalveolar Lavage Fluid

BALF was analyzed to determine total cell count, protein content (Bicinchoninic Acid Protein Assay Kit; Pierce, Rockford, IL, United States), and Lactic dehydrogenase (LDH) activity (LDH Detection Kit, Roche Applied Science, Indianapolis, IN, United States). The levels of TNF-α, IL-6 and CINC-1 in BALF were measured using commercial ELISA kits (R&D Systems Inc., Minneapolis, MN, United States), as instructed by the manufacturer.

### Determination of Malondialdehyde Level and Protein Carbonyl Content in Lung Tissues

The levels of MDA and protein carbonyl contents in right upper lung lobe ere determined as described previously (Liao et al., 2017; Wu et al., 2017) using the Carbonyl Content Assay Kit (Abcam, Cambridge, MA, United States) and Lipid Peroxidation (MDA) Assay Kit (Abcam) following the manufacturer’s instructions.

### Western Blotting

Prepared right middle lung lobe or cellular protein lysates containing 30 μg protein were separated by 10% SDS polyacrylamide gel electrophoresis and immunoblotting was performed as previously described (Liao et al., 2017; Hung et al., 2019). The membranes were probed with a β-actin antibody as a loading control (1:10,000; Sigma Chemical Company, St. Louis, MO, United States) and primary antibodies against B-cell lymphoma (Bcl)-2 (1:200; Santa Cruz Biotechnology, Dallas, Texas, United States), nuclear factor (NF)-κB (NF-κB) p65, phospho-NF-κB p65, inhibitor of NF-κB (IκB)-α, cleaved caspase-3 (1:1,000; Cell Signaling Technology, Danvers, MA, United States) or Lamin B1 (1:1,000; Abcam). All data are presented as the ratio of the target protein to the reference protein (β-actin).

### Immunohistochemical Analyses

Immunohistochemical staining to identify myeloperoxidase (MPO) and Ly6G was performed as previously described (Tang et al., 2019). Briefly, paraffin-embedded right lower lung lobe sections were deparaffinized, endogenous peroxidase activity was quenched using 3% H_2_O_2_ in 100% methanol for 15 min, and immunostaining was performed using the rabbit polyclonal antibody against MPO (1:100; Cell Signaling Technology) and rabbit polyclonal antibody against Ly6G (1:300, Biorbyt, UK).

### Pathological Evaluation

Paraffin sections of right lower lung lobe were stained with hematoxylin-eosin (H&E) to evaluate the extent of lung injury. The average number of polymorphonuclear neutrophils in the interstitium was determined from 10 different high-power fields (×400) by two investigators who were blinded to the groups. Semiquantitative grading of lung injury was performed as previously described (Wu et al., 2015; Liao et al., 2017). Briefly, within each field, lung injury was scored based on 1) infiltration or aggregation of neutrophils in the airspace or vessel wall, and 2) the thickness of the alveolar wall. Each assessment was graded on the following four-point scale: 0, 1, 2, or 3, for no, mild, moderate, or severe injury. The two scores were summed and recorded as the lung injury score for that section.

### Terminal Deoxynucleotidyl Transferase dUTP Nick End Labeling Assay of Lung Tissue

Paraffin-embedded lung tissue sections (5 mm-thick) were subjected to the TUNEL assay using the FragELTM DNA Fragmentation Detection Kit and Fluorescent-TdT Enzyme (Merck Millipore, Darmstadt, Germany) following the manufacturer’s instructions. TUNEL-positive nuclei were identified by fluorescence microscopy.

### Study Protocol

A total of 24 rat lungs were randomized to the following groups: control (0.9% NaCl, *n* = 6), P188 alone (1 mg/ml, *n* = 6), I/R alone (*n* = 6), or IR with P188 (1 mg/ml, *n* = 6). P188 was added to the reservoir containing 20 ml perfusate. IR was induced in the deflated lungs by stopping ventilation and perfusion for 40 min ischemia. After ischemia, perfusion and ventilation were continued for 60 min. The dose of P188 (Pluronic F-68; Sigma-Aldrich) was based on a previous study (Plataki et al., 2011).

### Cell Culture and Hypoxia/Reoxygenation Injury

Mouse alveolar type II epithelial (MLE-12) cells (American Type Culture Collection, Manassas, VA, United States) were cultured in DMEM/F-12 medium (Sigma-Aldrich) containing 10% fetal bovine serum, penicillin (100 U/mL), and streptomycin (10 μg/ml) in a humidified atmosphere containing 5% CO_2_ and 95% air (Pao et al., 2019). The cells were pretreated with saline, ammonium pyrrolidinedithiocarbamate 2 μM (PDTC, NF-κB specific inhibitor), or 1 mg/ml P188 (Plataki et al., 2011), then subjected to hypoxia (1% O_2_, 5% CO_2_, 94% N_2_) for 3 h, followed by reoxygenation (5% CO_2_, 95% air) for 2 h. Control cells were maintained under normoxic conditions without hypoxic stimulus. The cell supernatants were collected and assayed for chemokine (C-X-C motif) ligand 1 (CXCL1) using a mouse CXCL1 ELISA kit (R&D, Inc., Minneapolis, MN, United States).

### Measurement of Hydrogen Peroxide, Superoxide Dismutase and Glutathione in the Lung Tissue and Cells

The GSH levels were assessed using fluorometric glutathione detection assay kit (ab 65322, Abcam, Cambridge, MA, United States). H_2_O_2_ concentrations were measured using Hydrogen Peroxide Assay Kit (ab102500, Abcam). SOD activity was determined using a colorimetric Superoxide Dismutase Activity Assay Kit (ab65354; Abcam). All experiments were performed following the instructions of the manufacturer with each kit.

### Assessment of Cell Membrane Integrity Using Propidium Iodide

Propidium iodide (PI) is an impermeable nucleic acid dye that emits bright red fluorescence when it binds to DNA and RNA following cell membrane injury.

Following HR, the cells were stained with 200 μL of PI (1 mg/ml solution of PI diluted 1:3,000 in PBS) for 30 min, washed extensively in PBS for 3 min, fixed with 4% paraformaldehyde for 20 min, counterstained with 4′,6-diamidino-2-phenylindole (DAPI) to identify nuclei, and imaged using a fluorescence microscope.

### Measurements of Mitochondrial Membrane Potential (MMP)

The MMP of MLE-12 cells was assessed using the JC-1 Mitochondrial Membrane Potential Assay Kit (Abcam) according to the manufacturer's instructions. Briefly, the cells were incubated with 10 μmol/L JC-1 dye for 20 min at 37°C, washed twice with 1× dilution buffer, and imaged using a fluorescence microscope at excitation/emission wavelengths of 535/595 nm (green) and 485/535 nm (red).

### Cell Wounding and Repair Assays

MLE-12 cells were preincubated in medium with or without P188 (1 mg/ml) before HR. Fluorescent dextran (FDx, 2.5 mg/ml; Sigma-Aldrich) was added and monolayers were then exposed to HR. Cells were allowed to repair for 2 min, and then washed and incubated with PI-containing medium. The number of FDx- and PI-positive cells per × 20 view field was counted. Cells with green cytoplasmic dextran fluorescence were considered wounded but healed, whereas cells with red PI-fluorescent nuclei were considered wounded but permanently injured. The percentage of wounded and repaired cells was presented (Plataki et al., 2011).

### Data Analysis

Data are presented as mean ± SD and were analyzed using GraphPad Prism 5 for Windows (GraphPad Software, San Diego, CA, United States). Multiple group comparisons were performed using one-way ANOVA and the *post-hoc* Bonferroni test. LWG and PAP were assessed using repeated-measures two-way ANOVA followed by the *post-hoc* Bonferroni test. Statistical significance was defined as a *p*-value of 0.05 or less.

## Results

### Poloxamer 188 Reduces the Severity of Ischemia/Reperfusion-Induced Pulmonary Edema

**FIGURE 1:**
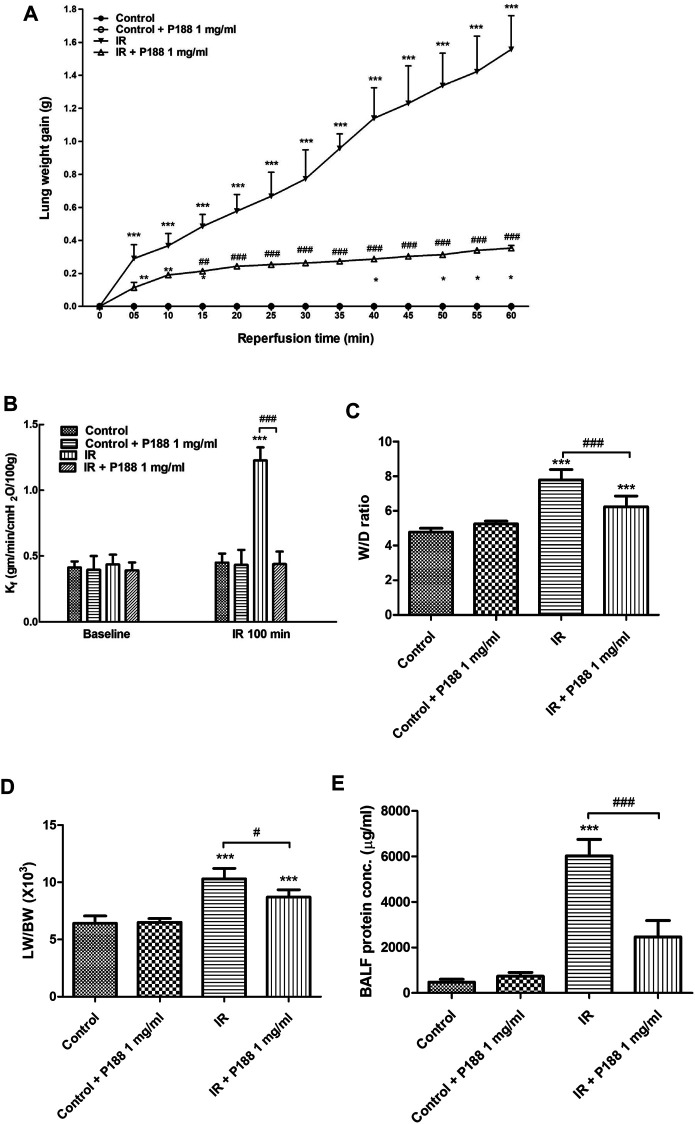
Effect of P188 on indices of pulmonary edema. Lung weight gain was determined continuously over 60 min reperfusion **(A)**. K_f_ **(B)**, lung wet weight/dry weight (W/D) ratio **(C)**, lung weight/body weight (LW/BW) ratio **(D)**, and protein concentration in bronchoalveolar lavage fluid (BALF) **(E)** were measured after 60 min reperfusion. Data are mean ± SD (6 rats per group); ***p < 0.001, compared with the control group; #*p* < 0.05, ##*p* < 0.01, ###*p* < 0.001, compared with the IR group.

IR induced a significant increase in lung weight over 60 min; however, P188 treatment suppressed this effect (Figure 1A). IR also led to a significantly higher K_f_, W/D ratio, LW/BW ratio, and concentration of protein in bronchoalveolar lavage fluid (BALF) after 60 min reperfusion (*p* < 0.05, Figures 1B–E). P188 treatment significantly reduced all these IR-induced increases in a dose-dependent manner.

### Poloxamer 188 Suppresses the Increase in PAP (ΔPAP) in Ischemia/Reperfusion-Induced Lung Injury

PAP remained steady during the 100 min observation period in the control group. IR led to a sudden, initial increase in PAP, and PAP subsequently decreased after reperfusion (Supplementary Figure 1). In lungs exposed to IR, PAP was significantly higher at 60 min after reperfusion than at baseline. However, treatment with P188 significantly suppressed the IR-induced increase in PAPin a dose-dependent manner (*p* < 0.05; Supplementary Figure 1).

### Poloxamer 188 Suppresses the Increases in Tumor Necrosis Factor-α, Cytokine-Induced Neutrophil Chemoattractant-1 and Interleukin-6 Levels, LDH Activity, and Total Cell Counts in Bronchoalveolar Lavage Fluid During Ischemia/Reperfusion-Induced Lung Injury

**FIGURE 2:**
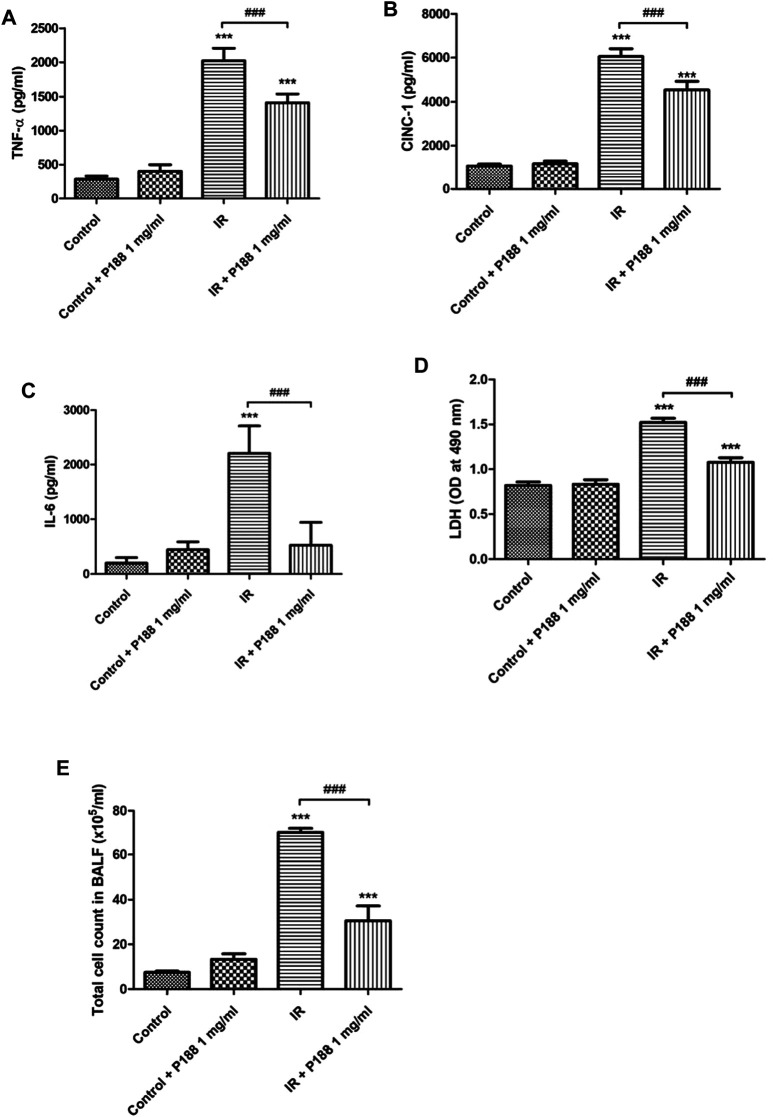
Effect of P188 on TNF-α, IL-6, and CINC-1 concentrations, LDH activity, and total cell count in BALF. Concentrations of TNF-α **(A)**, CINC-1 **(B)** and IL-6 **(C)**, LDH activity **(D)**, and total cell count **(E)** were measured after 60 min reperfusion. Data are mean ± SD (6 rats per group); ***p < 0.001 compared with the control group; ###*p* < 0.001 compared with the IR group.

IR significantly increased the concentrations of TNF-α, CINC-1, and IL-6*,* LDH activity, and the total cell count in BALF after 60 min reperfusion (*p* < 0.05; Figure 2). However, P188 significantly attenuated these IR-induced effects.

### Poloxamer 188 Attenuates the Increases in the Oxidative Stress in Ischemia/Reperfusion Lung Tissue

**FIGURE 3:**
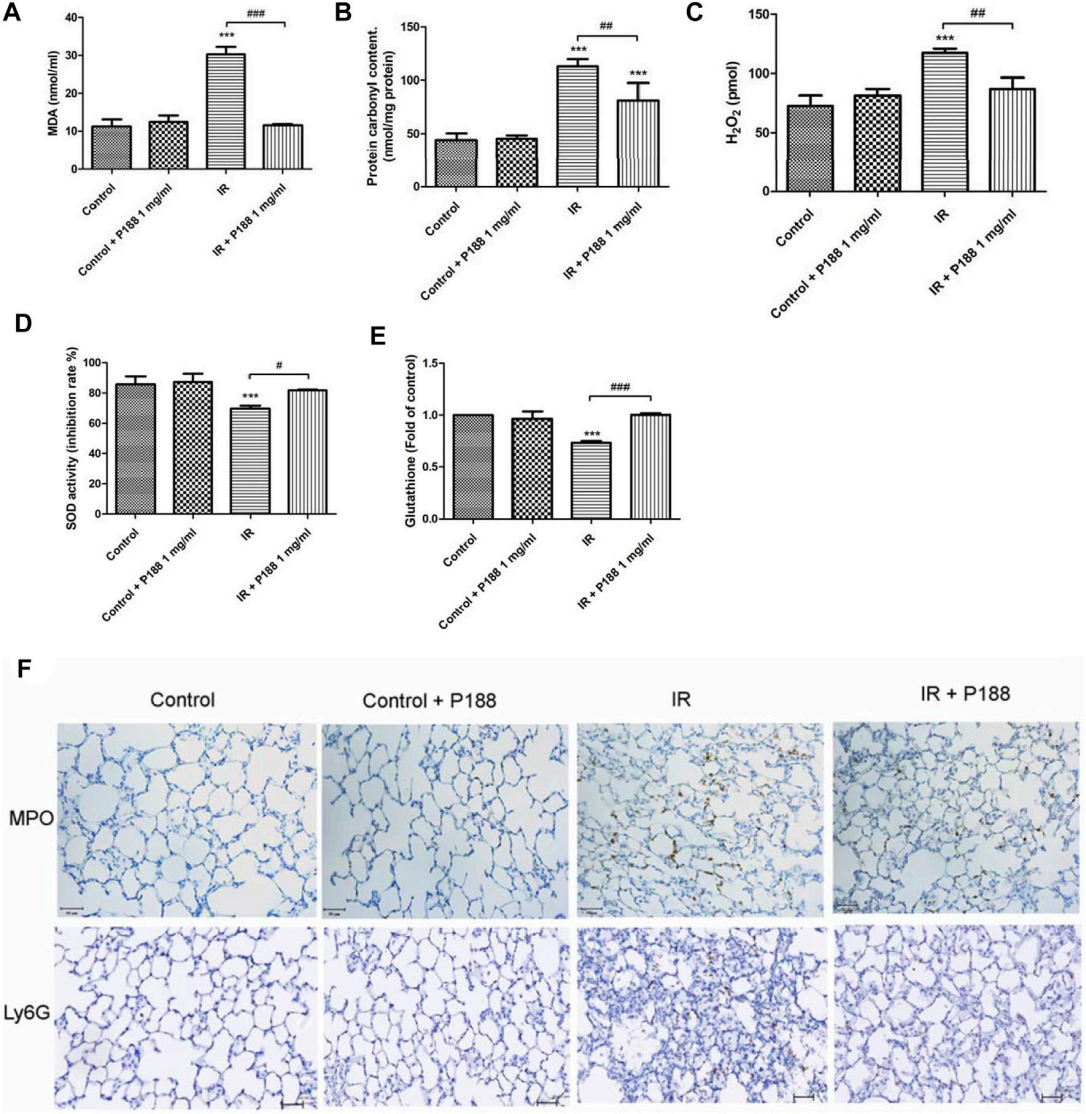
Effect of P188 on oxidative stress and the number of MPO and Ly6G -positive cells in lung tissues. Quantification of the MDA level **(A)**, carbonyl content **(B)**, H_2_O_2_ concentrations **(C)**, SOD activity **(D)**, and GSH levels **(E)** in the lung tissue were performed after 60 min reperfusion. **(F)** Immunohistochemical stainings of MPO and Ly6G were performed after 60 min reperfusion. Representative images (×200 magnification) are shown. Scale bar = 50 µm. Data are mean ± SD (6 rats per group); ***p < 0.001 compared with the control group; #*p* < 0.05, ##*p* < 0.01, ###*p* < 0.001 compared with the IR group.

IR significantly increased MDA level, protein carbonyl content, the production of H_2_O_2_, and decreased SOD activity and GSH levels in the lung tissues after 60 min reperfusion (*p* < 0.05, Figures 3A–E). However, treatment with P188 (1 mg/ml) significantly suppressed these IR-induced effects.

### Poloxamer 188 Attenuates the Increases in Neutrophil Infiltration in Ischemia/Reperfusion Lung Tissue

IR significantly increased the number of MPO and Ly6G (neutrophil marker) -positive cells in the lung tissues after 60 min reperfusion. However, treatment with P188 (1 mg/ml) significantly suppressed these IR-induced effects (Figure 3F).

### Poloxamer 188 Attenuates Histopathological Changes in Ischemia/Reperfusion Lung Tissue

**FIGURE 4:**
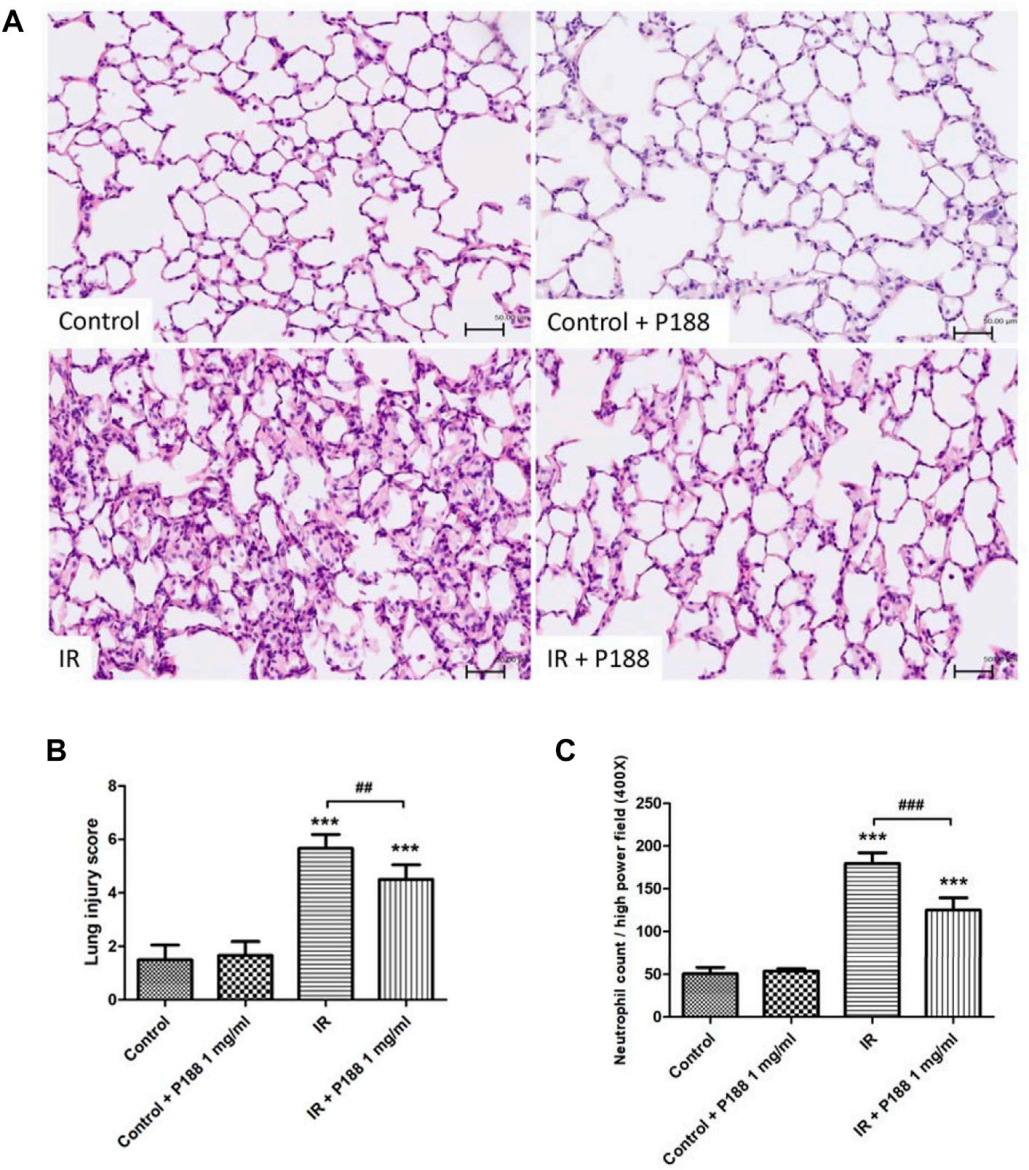
Effect of P188 on histopathological changes in lungs exposed to ischemia/reperfusion (IR). Hematoxylin and eosin staining (×200) **(A)**, lung injury scores **(B)**, and the number of neutrophils per high power field (×400 magnification) **(C)** were determined after 60 min reperfusion. Scale bar = 50 µm. Data are mean ± SD (6 rats per group). ***p < 0.001 compared with the control group; ##*p* < 0.01; ###*p* < 0.001 compared with the IR group.

IR induced histological abnormalities in the lung tissues, including enhanced neutrophil infiltration and obvious widening of the interalveolar walls. Treatment with P188 (1 mg/ml) attenuated the severity of these abnormalities after 60 min reperfusion (Figure 4A). Furthermore, treatment with P188 (1 mg/ml) also significantly lessened the lung injury scores (Figure 4B) and neutrophil infiltration (Figure 4C) in lungs exposed to IR.

### Poloxamer 188 Inhibits DNA Fragmentation, Cleaved Caspase-3, and Downregulation of Bcl-2 in Ischemia/Reperfusion Lung Tissue

**FIGURE 5:**
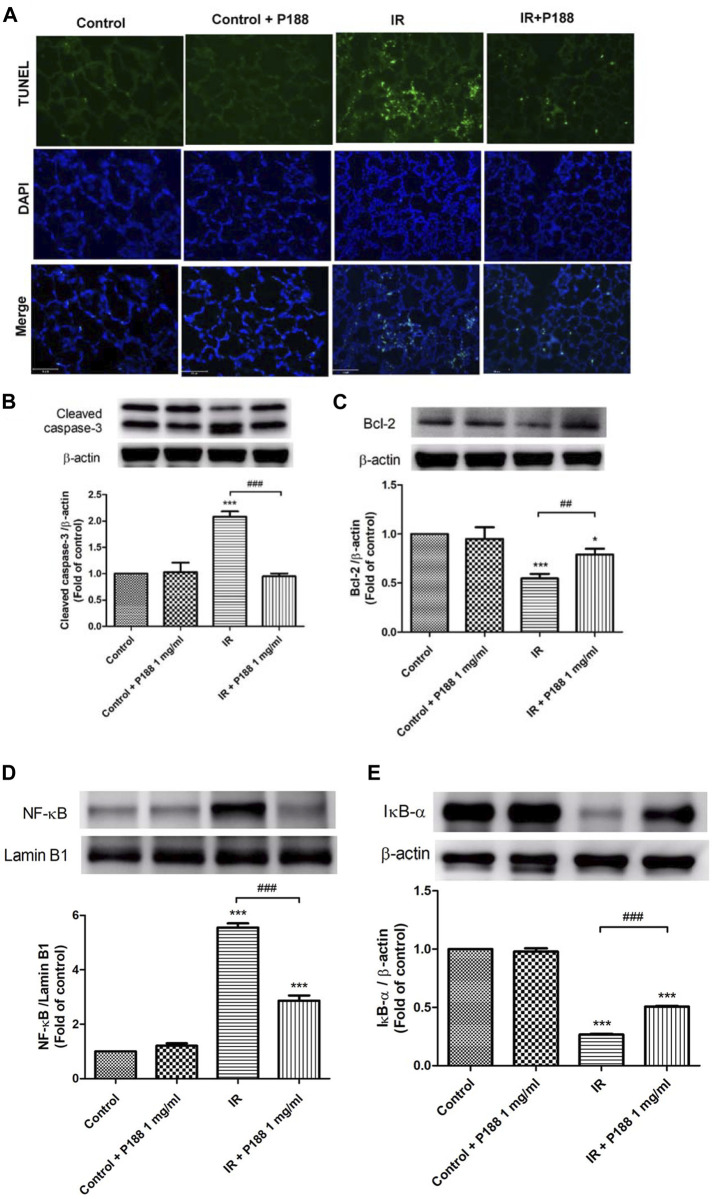
Effect of P188 on DNA fragmentation, expression of cleaved caspase-3 and Bcl-2, and NF-κB activation during ischemia/reperfusion (IR)-induced lung injury. **(A)** TUNEL assay of lung tissue. **(B–E)** Western blot analysis of cleaved caspase-3 **(B)**, Bcl-2 **(C)**, nuclear NF-κB p65 **(D)**, and cytoplasmic IκB-α **(E)** protein expressions in lung tissues. Lamin B1 and β-actin were used as loading controls for nuclear and cytoplasmic proteins, respectively. Representative blots are shown. Data are mean ± SD (6 rats per group); *p < 0.05, ***p < 0.001 compared with the control group; ##*p* < 0.05, ###*p* < 0.001 compared with the IR group.

The number of TUNEL-positive cells (Figure 5A) and the protein levels of cleaved caspase-3 (Figure 5B) were significantly higher, and the protein level of Bcl-2 was significantly lower (Figure 5C) in the lungs of the IR group after 60 min reperfusion compared to the control group. However, treatment with P188 significantly attenuated the severity of these apoptosis-related changes in lungs exposed to IR.

### Poloxamer 188 Attenuates Activation of the NF-κB Pathway in Ischemia/Reperfusion Lung Tissue

Western blot analysis of lung tissues indicated that IR significantly increased the nuclear level of NF-κB p65 and significantly decreased the cytoplasmic level of IκB-α after 60 min reperfusion (Figures 5D,E), indicating that IR activated the NF-κB pathway. However, treatment with P188 (1 mg/ml) significantly inhibited these effects.

### Poloxamer 188 Attenuates Hypoxia/Reoxygenation Injury in MLE-12 Cells

**FIGURE 6:**
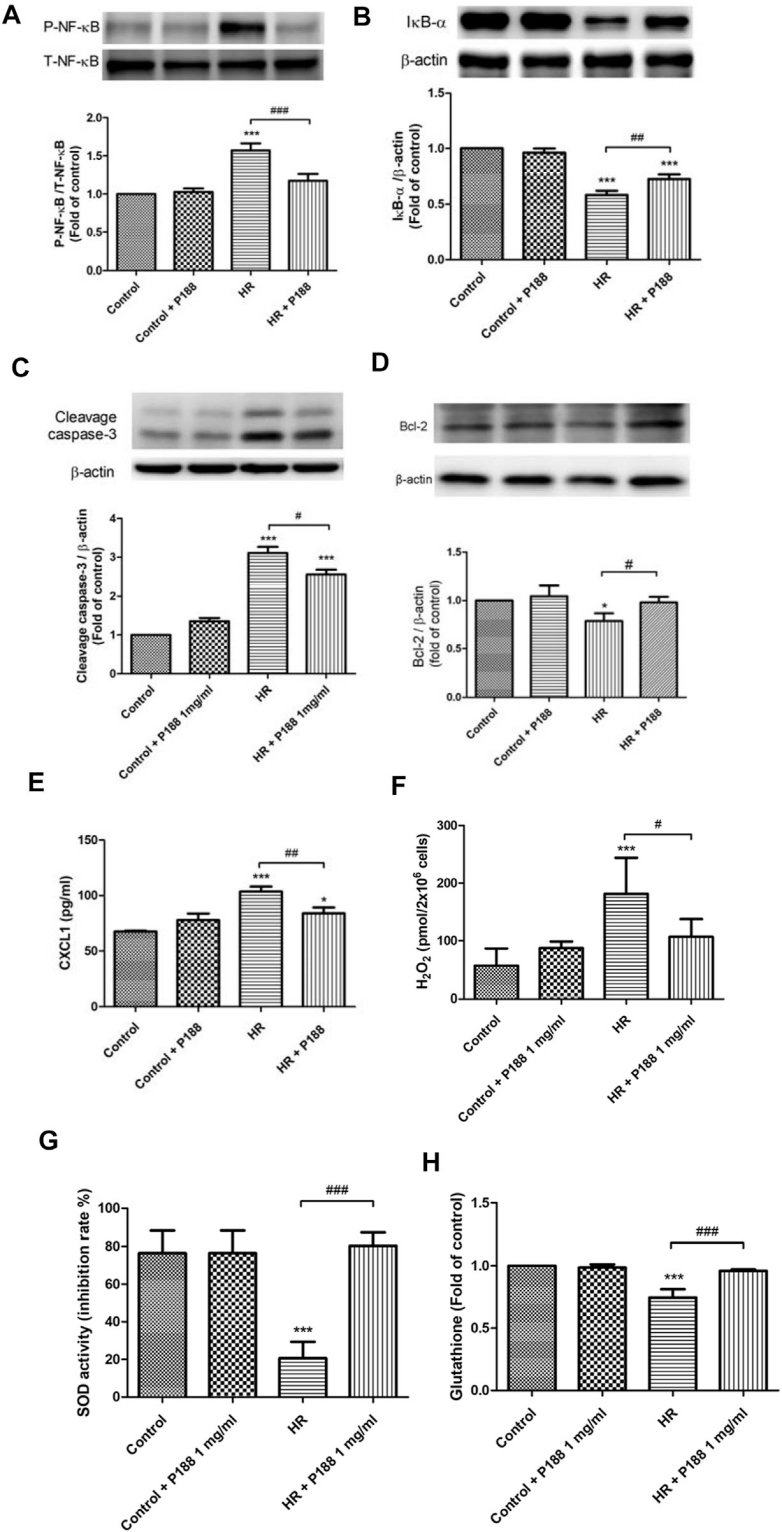
Effect of P188 on hypoxia/reoxygenation (HR) injury in MLE-12 cells. NF-κB phosphorylation **(A)**, IκB-α degradation **(B)**, cleaved caspase-3 **(C)** and Bcl-2 **(D)** protein expression, CXCL-1 **(E)**, hydrogen peroxide (H_2_O_2_) **(F)**, superoxide dismutase (SOD) **(G)**, and glutathione (GSH) **(H)** levels were measured at 2 h after HR. β-actin served as the loading control. A representative blot is shown. Data are mean ± SD (*n* = 6 per group); *p < 0.05, ***p < 0.001 compared with the control group. #*p* < 0.05, ##*p* < 0.01, ###*p* < 0.001 compared with the HR group.

The protective effects of P188 on HR injury were examined using MLE-12 cells (Figure 6). HR increased phosphorylation of NF-κB and cleaved caspase-3 protein expression, decreased IκB-α and BCL-2 protein expression, and upregulated CXCL-1 levels at 2 h after H/R (Figures 6A–E). However, P188 (1 mg/ml) significantly inhibited these HR-induced effects.

### Poloxamer 188 Attenuates Oxidative Stress in MLE-12 Cells Exposed to Hypoxia/Reoxygenation

HR increased the production of H_2_O_2_, and decreased SOD activity and GSH levels at 2 h after HR. However, P188 (1 mg/ml) significantly inhibited these HR-induced effects (Figures 6F–H).

### PDTC Reduces Hypoxia/Reoxygenation Injury in MLE-12 Cells

The protective effects of PDTC on HR injury were examined using MLE-12 cells (Supplementary Figure 2). PDTC decreased phosphorylation of NF-κB, increased IκB-α expression, and reduced CXCL-1 levels at 2 h after HR (Supplementary Figures 2A–D).

### Poloxamer 188 Attenuates the Depolarization of MMP in MLE-12 Cells Exposed to Hypoxia/Reoxygenation

**FIGURE 7:**
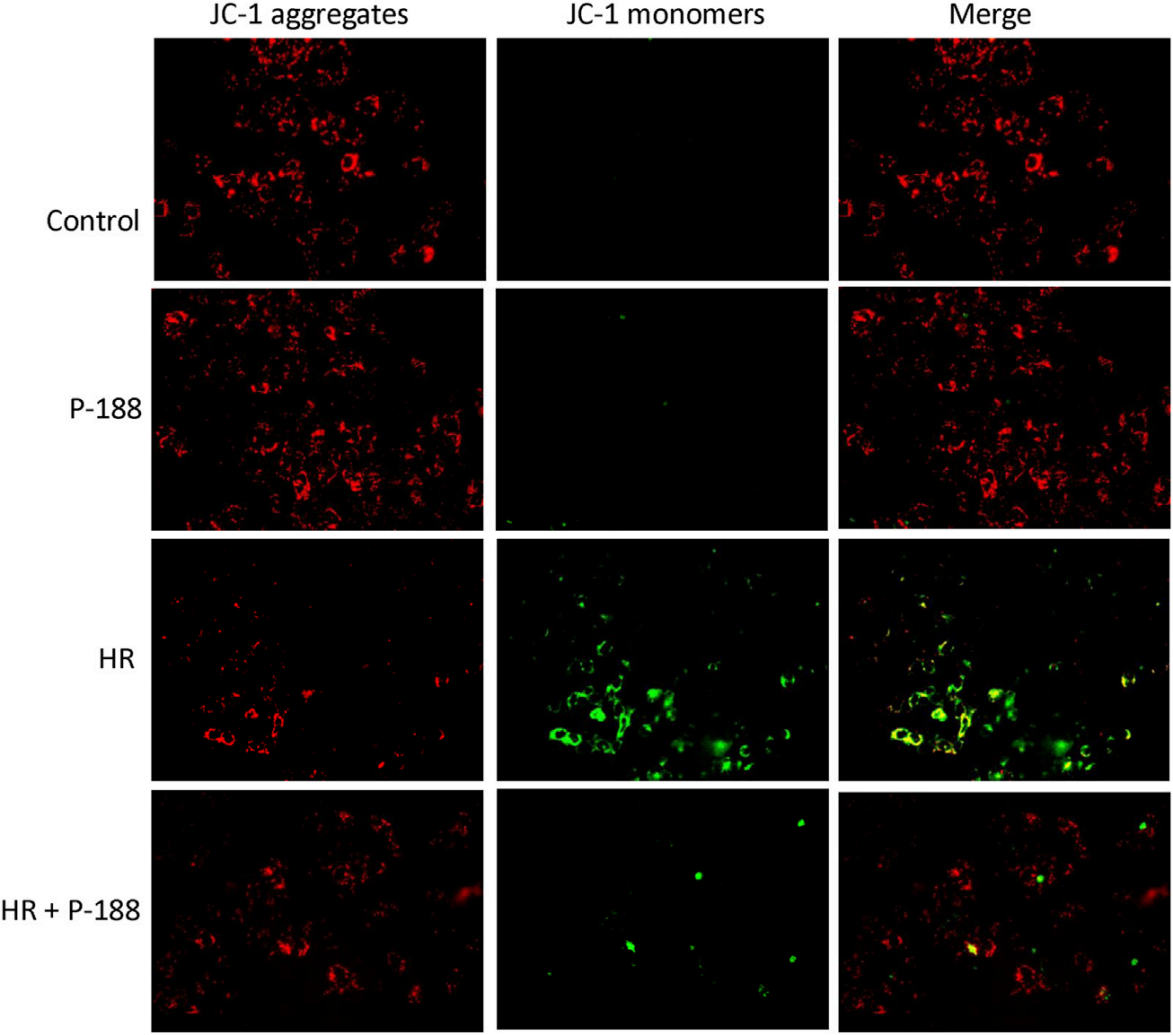
Effect of P188 on depolarization of mitochondrial membrane potential in MLE-12 cells exposed to hypoxia/reoxygenation (HR). Red JC-1 dimers indicate normal mitochondrial membrane potential. Green JC-1 monomers reflect depolarization of mitochondrial membrane potential. The red dimers and green monomers co-localized. Experiments were repeated three times each.

MMP was determined using the probe JC-1, which easily enters cells and normal mitochondria. At high MMP, JC-1 forms red fluorescent aggregates. At low MMP, JC-1 is present as a green fluorescent monomer. JC-1 exhibited red fluorescence in control MLE-12 cells, indicating normal MMP. Exposure of MLE-12 cells to HR resulted in MMP depolarization, as indicated by increased levels of green fluorescence in JC-1 stained cells. However, P188 treatment blocked HR-induced MMP depolarization, as confirmed by higher levels of red fluorescence and lower levels of green fluorescence compared to cells exposed to HR alone (Figure 7).

### Poloxamer 188 Maintains Cell Membrane Integrity in MLE-12 Cells Exposed to Hypoxia/Reoxygenation

**FIGURE 8:**
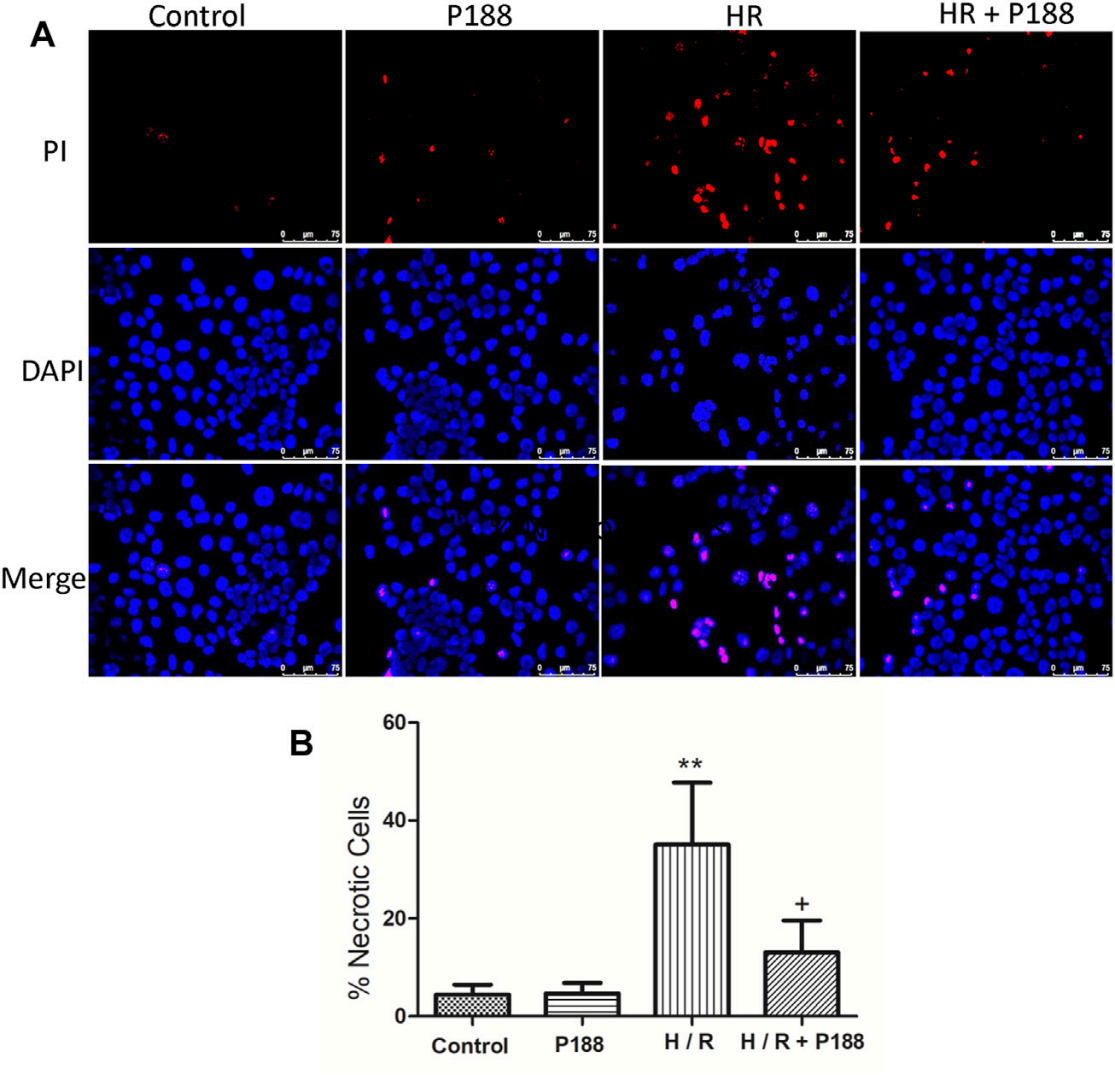
P188 protects against hypoxia/reoxygenation (HR)-induced MLE-12 cell death. **(A)** Representative fluorescence images of MLE-12 cells stained with propidium iodide (red color) and counterstained with DAPI (blue color). **(B)** Percentage of necrotic cells exposed to HR. Data are mean ± SD (*n* = 6 experiments under each condition). **p < 0.01 compared with the control group; #*p* < 0.05 compared with the HR group.

Propidium iodide (PI, red fluorescence) labelling was used to detect cell membrane disruption and DAPI counterstaining (blue fluorescence) was used to visualize cell nuclei. No obvious PI staining was detected in the control cells, whereas HR significantly increased the number of PI-positive cells (Figure 8). However, P188 reduced the number of PI-positive cells in cells exposed to HR.

### Poloxamer 188 Enhances Cell Membrane Repair After Hypoxia/Reoxygenation Injury in MLE-12 Cells

**FIGURE 9:**
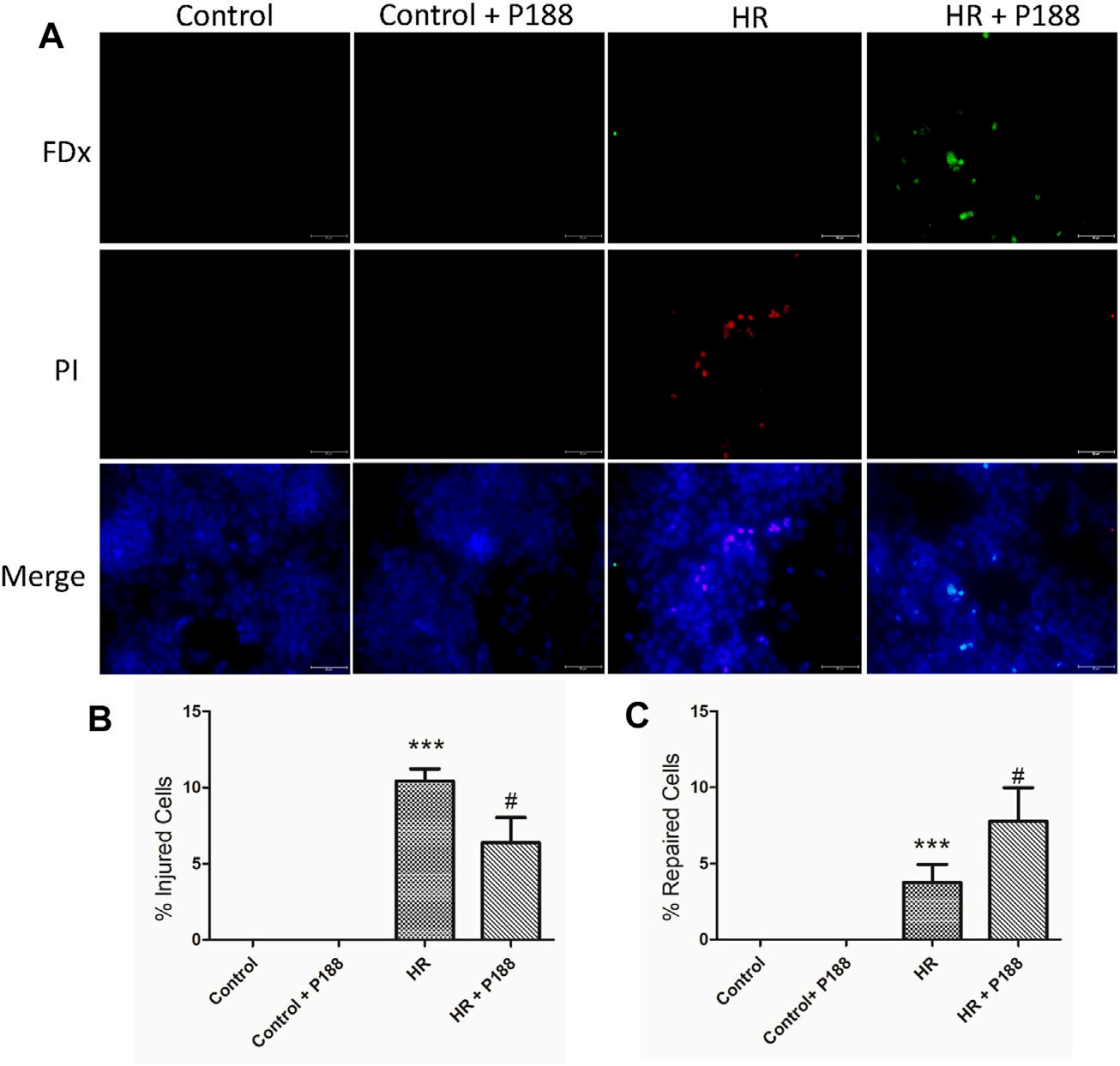
**(A)** P188 enhances cell membrane repair after hypoxia/reoxygenation (HR) injury in MLE-12 cells. Representative fluorescence images of cells exposed to HR in the presence or absence of P188. Cells with green cytoplasmic dextran (FDx) fluorescence were considered wounded but healed, whereas cells with red propidium iodide (PI) fluorescent nuclei were considered wounded but permanently injured. Images were merged with DAPI nuclear counterstain (blue). **(B)** Percentage of injured cells per field. **(C)** Percentage of repaired cells per field. Scale bar shows 100 μm. Data are mean ± SD (*n* = 6 plates). ***p < 0.001 compared with the control group; #*p* < 0.05 compared with the HR group.

As shown in Figure 9, in the presence of P188, there were significantly fewer wound fluorescently labeled cells (FDx and PI). The percentage of FDx-labeled cells relative to the total number of labeled cells was significantly greater in the HR + P188 group, indicating that more cells repaired when P188 was added.

## Discussion

This study demonstrates that administration of P188 exerts a protective effect against IR-induced injury in rat lungs. P188 decreased lung edema by reducing vascular permeability, PAP, the LW/BW and W/D lung weight ratios, LWG, and the protein levels and LDH activity in BALF. P188 treatment also suppressed IR-induced production of pro-inflammatory cytokines and free radicals, reduced the influx of pulmonary neutrophils, and attenuated apoptosis and tissue damage. In addition, P188 inhibited IR-induced activation of the NF-κB signaling pathway. *In vitro* experiments showed that P188 decreased the levels of phosphorylated NFκB p65 and CXCL-1, apoptosis, oxidative stress, and increased the level of IκB-α in MLE-12 cells exposed to HR. Furthermore, P188 inhibited MMP depolarization, maintained membrane integrity, and promoted cell repair *in vitro.* The ability of P188 to attenuate these abnormalities implies P188 may have potential as an adjunct treatment to reduce IR-induced lung injury during lung transplantation.

The protective ability of P188 to insert into membranes may represent an attractive strategy to maintain membrane integrity (Moloughney and Weisleder, 2012). We used PI to assess membrane integrity *in vitro*. When cell membrane integrity is disrupted, PI can penetrate into cells, bind to DNA and RNA and emit red fluorescence; thus, only necrotic cells produce red fluorescence. In the current study, P188 reduced the number of PI-positive cells following HR, indicating P188 maintained cell membrane integrity.

In addition to its membrane-resealing effects, the membrane surfactant P188 may also exert protective effects via other mechanisms. Firstly, under pathologic situations such as acute lung IR, epithelial barrier disruption alters pulmonary vascular permeability, increases penetration of proteins into alveolar regions, and leads to lung edema. Our results indicate that P188 prevented the increase in vascular permeability induced by IR. Similarly, P188 protected against increased vascular permeability in rats exposed to injurious ventilation and prevented extravasation of lung fluid in rats subjected to hemorrhagic shock (Zhang et al., 2009; Plataki et al., 2011).

P188 has been proven to exert anti-inflammatory effects in several experimental models, such as reperfusion injury during prolonged hypotensive resuscitation, acute myocardial infarction, and bleomycin toxicity (Justicz et al., 1991; Tan and Saltzman, 1999; Zhang et al., 2009). Oxidative stress is well-recognized to play a vital role in the pathogenesis of IR injury (Ferrari and Andrade, 2015; Hsu et al., 2015; Wu et al., 2015). Oxidative stress can induce epithelial and endothelial damage, increase vascular permeability, enhance neutrophil infiltration, and promote formation of edema in the lungs (Ferrari and Andrade, 2015). Numerous studies have indicated that inhibition of oxidative stress represents an effective therapeutic approach in animal models of IR lung injury (Bao et al., 2012; Ferrari and Andrade, 2015). Our results demonstrate that P188 can also reduce oxidative stress in IR lung tissues and MLE-12 epithelial cells exposed to HR. A previous investigation showed that P188 significantly blocked lipid peroxidation of hippocampal and cerebellar neurons induced by Fe^2+^ and H_2_O_2_ (Marks et al., 2001). In addition, P188 also decreased lipid peroxidation in the spinal cords of G93ASOD1 transgenic mice (Riehm et al., 2018). Insertion of the hydrophobic polypropylene block of P188 into cell membranes may possibly reduce lipid peroxidation (Inyang et al., 2020). Therefore, inhibition of lipid peroxidation may partly explain the ability of P188 to protect against IR-induced lung injury.

IR dramatically increases the number of rolling and adherent leukocytes, which are primarily neutrophils (Kalogeris et al., 2012; Laubach and Sharma, 2016; Liao et al., 2017). Neutrophils secrete a host of inflammatory mediators that contribute to tissue damage. Indeed, depletion of neutrophils significantly reduced IR-induced tissue injury (Kalogeris et al., 2012; Laubach and Sharma, 2016). In this study, P188 decreased neutrophil infiltration into IR lung tissues, as indicated by lower numbers of neutrophils and MPO-positive cells. These observations are comparable with reports that P188 reduced neutrophil migration and adherence, prevented neutrophil transfer to inflammatory sites, and attenuated the release of proteolytic enzymes from neutrophils in *in vitro* studies and animal models (Lane and Lamkin, 1984, 1986; Babbitt et al., 1990; Tan and Saltzman, 1999).

Apoptosis plays a crucial role in IR-induced lung injury (Ng et al., 2005). Membrane injury induced by IR can promote cell death. Disruption of membrane integrity initiates a variety of downstream events that result in secondary cellular injury. Conversely, inactivation of apoptotic signaling pathways can attenuate IR lung injury (Ng et al., 2005; Hsu et al., 2015; Liao et al., 2017). In the current study, P188 reduced the levels of apoptosis in the lung tissues after IR, as indicated by decreased expression of cleaved caspase-3, reduced numbers of TUNEL-positive cells, and increased Bcl-2 expression. P188 also significantly decreased the number of PI-positive epithelial cells and apoptosis after HR *in vitro*. Similarly, P188 markedly decreased the numbers of TUNEL-positive and PI-positive neuronal cells after mechanical and IR injury, reduced lysosomal membrane permeabilization-mediated cell apoptosis *in vitro* and *in vivo*, and decreased caspase activity induced by hemorrhagic shock (Serbest et al., 2005; Serbest et al., 2006; Zhang et al., 2009; Gu et al., 2013; Wang et al., 2017; Dong et al., 2019).

Assessment of mitochondrial membrane potential using the dual-emission dye JC-1 provides an earlier indicator of cell death than TUNEL or PI staining. Low mitochondrial potential was observed in MLE-12 epithelial cells exposed to HR. However, P188 attenuated the reduction in mitochondrial membrane potential in cells exposed to HR. Several investigators recently reported that P188 acts directly on the mitochondria to prevent mitochondrial outer membrane permeabilization, reduce mitochondrial dysfunction and inhibit mitochondrial-dependent death pathways in models of neuronal injury (Shelat et al., 2013; Luo et al., 2015; Wang et al., 2017). Thus, lung cells that do not directly break down in response to IR may subsequently undergo apoptosis. This study demonstrates that the ability of P188 to repair the initial membrane injury caused by IR not only prevented lung cells from acute death, but also inhibited the secondary events that lead to late cell death.

The transcription factor NF-κB modulates the production of various pro-inflammatory cytokines and chemokines. When IκB is degraded, active NF-κB translocates into the nucleus, and enhances the transcription of pro-inflammatory cytokines such as TNF-α and CINC-1, which exacerbate lung injury by inducing production of additional pro-inflammatory cytokines and promoting leukocyte infiltration. Previous experiments demonstrated that activation of NF-κB plays an important role in IR-induced lung injury (Hsu et al., 2015; Wu et al., 2015; Liao et al., 2017). This study clearly showed that P188 significantly suppressed IR-induced activation of NF-κB, which decreased the production of proinflammatory cytokines and reduced infiltration of leukocytes. Furthermore, investigation of the direct effects of P188 on alveolar epithelial cells showed that P188 significantly inhibited degradation of IκBα, phosphorylation of NF-κB p65, and the production of KC in MLE-12 epithelial cells subjected to HR. In addition, PDTC (NF-κB specific inhibitor) also provided similar effects in HR-exposed MLE-12 cells. These results corroborate a previous study, which showed that P188 suppressed the NF-κB signaling pathway in a mouse model of intracerebral hemorrhage (Wang et al., 2015).

The literature suggests that P188 exerts medical benefits in the treatment of various disorders. P188 is also widely available and easy to manufacture [2]. Numerous pharmacological approaches to reduce I/R lung injury have proven largely ineffective in clinical settings (Laubach and Sharma, 2016). The use of P188 may represent an alternative simple, safe therapeutic approach. To the best of our knowledge, this is the first study to show that P188 significantly attenuates IR-induced acute lung injury. We administered P188 at the onset of ischemia, thus P188 may be suitable as an adjunct during lung transplantation procedures. However, future studies are required to address the value of administering P188 after I/R has occurred.

In summary, this study demonstrates that P188 can protect against IR-induced lung injury *in vivo* and *in vitro* via mechanisms involving maintenance of plasma membrane integrity and inhibition of multiple signaling pathways. P188 has potential as an effective adjunct to reduce IR-induced injury during lung transplantation.

## Data Availability

The original contributions presented in the study are included in the article/Supplementary Material, further inquiries can be directed to the corresponding author.

## Supplementary Material

Supplementary Figure 1 Effect of P188 on pulmonary artery pressure (ΔPAP). Change in pulmonary artery pressure (ΔPAP) from baseline was measured continuously during 60 min reperfusion. Data are mean ± SD (6 rats per group); ****p* < 0.001 compared with the control group; ###*p* < 0.001 compared with the IR group.

Supplementary Figure 2 Effect of ammonium pyrrolidinedithiocarbamate (PDTC) on hypoxia/reoxygenation (HR) injury in MLE-12 cells. NF-κB phosphorylation, IκB-α degradation, and CXCL-1level were measured at 2 h after HR. NF-κB was detected using **(A)** immunofluorescence staining and **(B)** Western blotting. β-actin served as the loading control. Representative blot and image are shown. Data are mean ± SD (*n* = 6 per group); **p* < 0.05, ***p* < 0.01,****p* < 0.001 compared with the control group. #*p* < 0.05, ##*p* < 0.01, ###*p* < 0.001 compared with the HR group.
