# Supplementary figures and images for: Poloxamer 188 Attenuates Ischemia-Reperfusion-Induced Lung Injury by Maintaining Cell Membrane Integrity and Inhibiting Multiple Signaling Pathways
Source: Front Pharmacol. 2021 Jul 15;12:650573. doi: 10.3389/fphar.2021.650573 (PMC8319770; doi:10.3389/fphar.2021.650573)

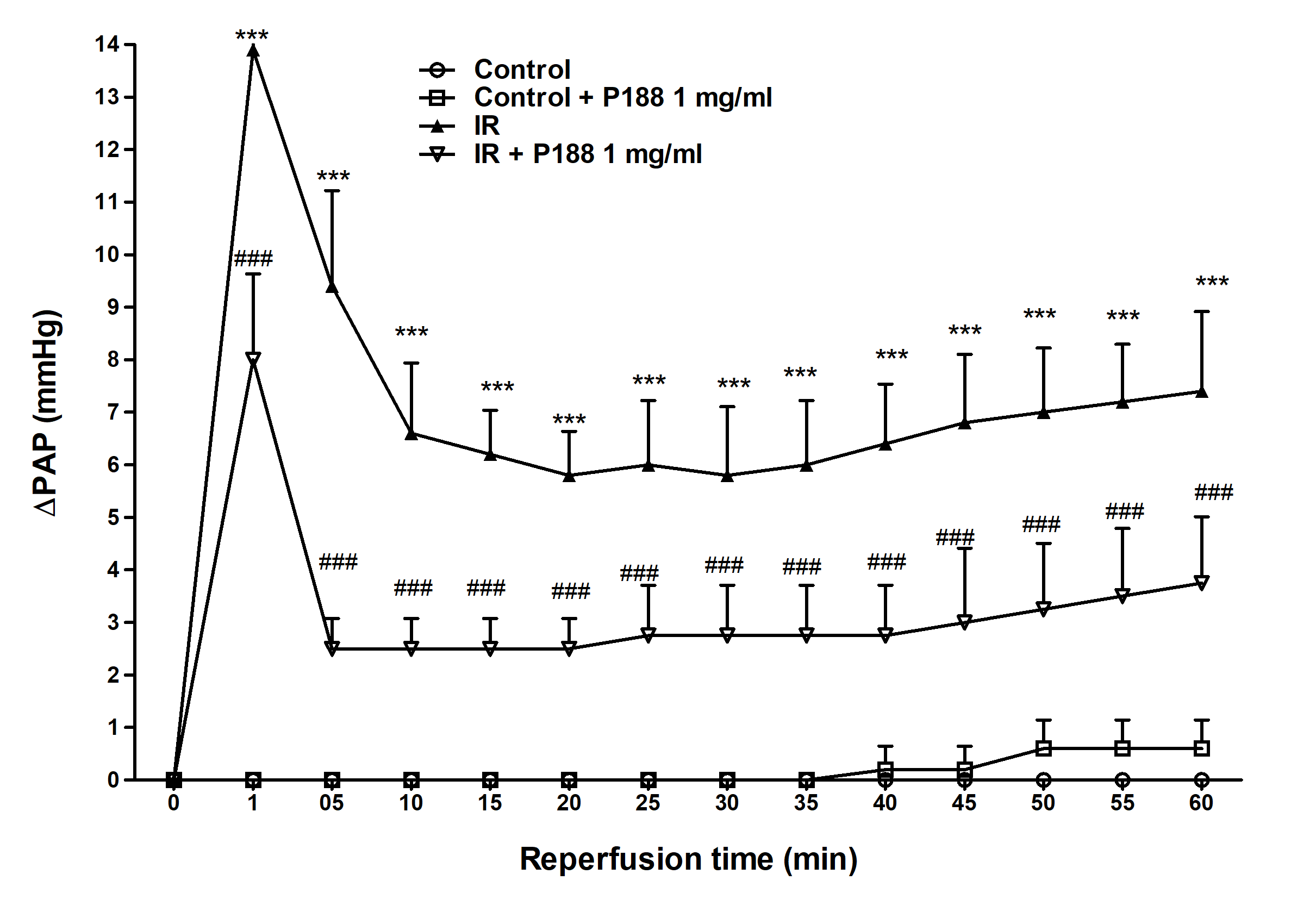

Supplement: Supplementary Figure 1 — Effect of P188 on pulmonary artery pressure (ΔPAP). Change in pulmonary artery pressure (ΔPAP) from baseline was measured continuously during 60 min reperfusion. Data are mean ± SD (6 rats per group); ***p < 0.001 compared with the control group; ###p < 0.001 compared with the IR group. [file Image1.TIF]

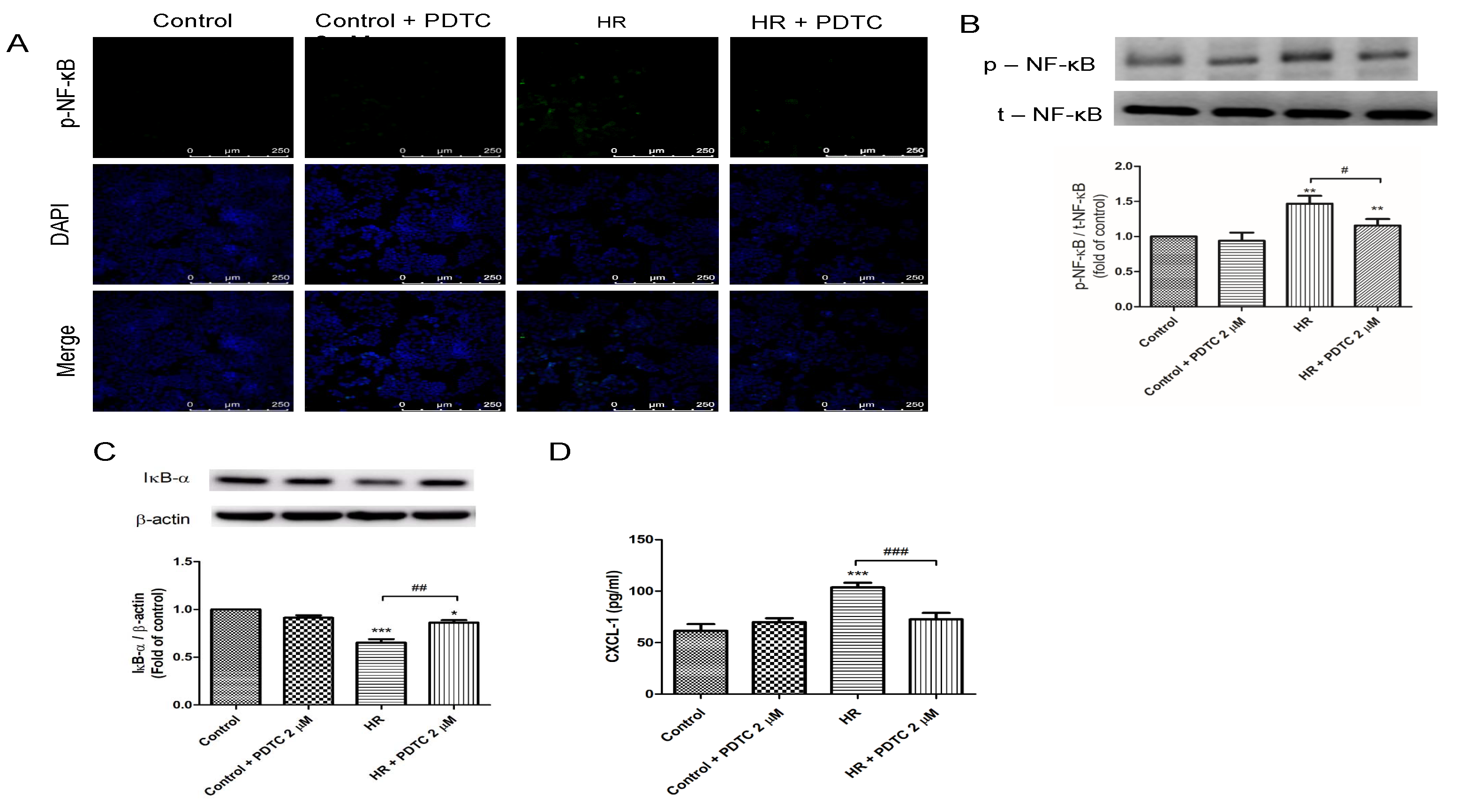

Supplement: Supplementary Figure 2 — Effect of ammonium pyrrolidinedithiocarbamate (PDTC) on hypoxia/reoxygenation (HR) injury in MLE-12 cells. NF-κB phosphorylation, IκB-α degradation, and CXCL-1level were measured at 2 h after HR. NF-κB was detected using (A) immunofluorescence staining and (B) Western blotting. β-actin served as the loading control. Representative blot and image are shown. Data are mean ± SD (n = 6 per group); *p < 0.05, **p < 0.01,***p < 0.001 compared with the control group. #p < 0.05, ##p < 0.01, ###p < 0.001 compared with the HR group. [file Image2.TIF]
